# Deworming and Development: Asking the Right Questions, Asking the Questions Right

**DOI:** 10.1371/journal.pntd.0000362

**Published:** 2009-01-27

**Authors:** Donald A. P. Bundy, Michael Kremer, Hoyt Bleakley, Matthew C. H. Jukes, Edward Miguel

**Affiliations:** 1 Human Development Network, World Bank, Washington, D.C., United States of America; 2 Department of Economics, Harvard University, Cambridge, Massachusetts, United States of America; 3 Graduate School of Business, University of Chicago, Illinois, United States of America; 4 Graduate School of Education, Harvard University, Cambridge, Massachusetts, United States of America; 5 Center of Evaluation for Global Action, University of California, Berkeley, California, United States of America; *PLoS Neglected Tropical Diseases*, United States of America

Two billion people are infected with intestinal worms [1]. In many areas, the majority of schoolchildren are infected, and the World Health Organization (WHO) has called for school-based mass deworming. The key area for debate is not whether deworming medicine works—in fact, the medical literature finds that treatment is highly effective [2], and thus the standard of care calls for treating any patient known to harbor an infection. As the authors of the Cochrane systematic review point out, a critical issue in evaluating current soil-transmitted helminth policies is whether the benefits of deworming exceed the costs or whether it would be more prudent to use the money for other purposes [3].

While in general we think the Cochrane approach is very valuable, we argue below that many of the underlying studies of deworming suffer from three critical methodological problems: treatment externalities in dynamic infection systems, inadequate measurement of cognitive outcomes and school attendance, and sample attrition. We then argue that the currently available evidence from studies that address these issues is consistent with the consensus view expressed by other reviews and by policymakers that deworming is a very cost-effective way to increase school participation and has a high benefit to cost ratio.

## Treatment Externalities

Most of the studies included in David Taylor-Robinson and colleagues' systematic review do not adequately address the population dynamics of helminth infection. These studies follow standard practice in clinical trials and consider untreated people as a control group. But geohelminth transmission is a dynamic process, and both theoretical and community studies have shown that treatment of some individuals leads to a reduction in transmission in the community as a whole [4],[5]. Thus, in a trial randomized at the level of the individual, the expected difference between treatment and control children within the same area will be less than the actual treatment effect. If, for example, school attendance increases by 8 percentage points among treated children and by 4 percentage points among the untreated due to externalities, the estimated impact using this technique will only be 4 percentage points, rather than the true effect of 8 percentage points. These concerns are not merely hypothetical: a study in Kenya found large health and educational spillovers to untreated students within treated schools and even to students in nearby schools [6]. In light of this finding, the primary focus of a review should be studies that use a cluster design and correct standard errors for intra-cluster correlation [6]–[8], if indeed the purpose of such a review is to evaluate the desirability of mass deworming as a policy. The three studies cited which used this approach, some of which were excluded from the Cochrane review, did find positive effects of deworming.

## Measuring Cognitive Outcomes and School Attendance

The summary of the Cochrane review [3] published in this issue of *PLoS Neglected Tropical Diseases* focuses on biomedical outcomes while only touching on cognitive and educational issues in a single paragraph.

Measuring the impact of a health intervention on cognitive outcomes requires careful consideration based on an understanding of the nature of cognitive development, and at least three issues need to be addressed [9]. First, impaired cognition rarely results from a single cause [10]. Worm infections are likely to affect children's cognitive development differently according to their levels of poverty, psychosocial stimulation, and general health status. Reporting of these other environmental risk factors is essential for interpreting studies on cognitive impacts, yet such reporting is rarely used as an inclusion criterion in systematic reviews. Second, the cumulative and interacting impacts of multiple threats to cognitive development typically means a range of functions could be affected, requiring a comprehensive battery of cognitive assessments. However, Taylor-Robinson and colleagues did not give the design of these cognitive assessments the same weight as other methodological considerations when selecting studies for their systematic review. Finally, recovery of cognitive impairments may depend on remedial education or psychosocial stimulation in addition to treatment of the disease leading to the impairment [11]. Consequently, null results with cognitive outcomes are difficult to interpret unless trial designs address the above issues.

When measuring the quantity of schooling, it is also critical to directly verify attendance through independent checks on site rather than relying on reported data, which is often influenced by incentives for teachers to exaggerate enrollment and attendance to increase funding. One study found large discrepancies between school attendance measured by registers versus spot checks in a sample of Kenyan primary schools, with average attendance over 10 percentage points higher in the school registers data [6]. The two trials included in the review reported negative findings on attendance [12],[13], but both relied on such secondary data.

## Sample Attrition

The impact of deworming on school participation creates its own methodological problem of sample attrition, which was not adequately addressed in the studies that were included in the Cochrane review [3]. For example, one included study reports test score data for 89% of students in the treatment group but only 59% in the comparison group [14]. If fewer test scores are available for pupils in the comparison group because academically marginal pupils are more likely to be absent, then the true impact of deworming will be underestimated. This attrition bias might also explain why another study found no effect of deworming on primary school attendance after excluding all periods of extended school absence, perhaps the very effect they were seeking to detect [13].

## Evidence on Health and Education

Even without addressing the concerns about treatment externalities, the Cochrane systematic review found that “[w]eight gain after one dose of anthelminth drugs became just significant, and with confidence intervals that include potentially important weight gain values” [3]. This is despite the notorious difficulty of detecting change in growth in school-age children. Another recent systematic review found that deworming shows a small effect on anemia where worm infection is common [15], and another concludes that “all (included) studies showed a benefit [of deworming] for maternal and child health” [16].

A large school-based study addressing the above methodological issues found that treatment reduced absence by 7 percentage points, amounting to a 25 percent decline in total absence [6]. (Note that contrary to the claim in the Cochrane systematic review, the results in [6] are not confounded by uncontrolled use of praziquantel. The school participation benefits of deworming are similarly large and statistically significant in the study subregion, consisting of 58 primary schools, where schistosomiasis was largely absent and where the protocol thus called for praziquantel not to be provided.)

## Costs and Benefits

From an economic policy perspective, the merits of deworming depend mainly on whether its long-term impact on earnings exceeds its cost. Deworming costs pennies per dose, or about US$0.25 per child per year with delivery costs, so gains of a mere fraction of a percent in income would provide a very high benefit to cost ratio. Studies designed to pick up such effect sizes would have to be large and long-lived, perhaps prohibitively so in the setting of a randomized controlled trial. Fortunately, history provides a natural experiment—the Rockefeller-sponsored campaign against hookworm in the United States South in the 1910s. Census data and difference-in-difference analysis have been used to examine the interaction effect of the pre-campaign prevalence of hookworm in different parts of the South with the timing of a mass deworming program [17]. The study found large gains in literacy, school attendance, and subsequent income among cohorts offered deworming as children, implying that persistent hookworm infection in childhood depressed eventual educational attainment by 2.1 years and adult income by 40%. The findings imply that worms accounted for 22% of the large 1900 income gap between the US South and North. Based on the estimated rate of return to education in Kenya, deworming is likely to increase the net present value of wages by over US$30 per treated individual, creating a benefit to cost ratio of over 100. Even if these estimates from Kenya and the US South [17] overstate the economic returns by an order of magnitude, the benefit to cost ratio would be highly favorable.

## Conclusions

Existing evidence indicates that mass school-based deworming is extraordinarily cost-effective once health, educational, and economic outcomes are all taken into account, and it is thus unsurprising that a series of studies from the 1993 World Development Report [18] to the recent Copenhagen Consensus [19] argue that treatment of the most prevalent worm infections is a very high return investment. A review by the Abdul Latif Jameel Poverty Action Lab at the Massachusetts Institute of Technology found that deworming was by far the most cost-effective way to increase primary school participation [20]. These analyses depend in part on the impact of deworming on the biomedical outcomes that are the focus of the Cochrane systematic review [3], but they also depend on the implications for the future development of the individual and society. Future income is a central measure of this development. Because there is strong evidence that obtaining more education leads to higher adult income, the effect of deworming on school participation should be central to any reasonable policy analysis.

We believe that future iterations of the Cochrane review that address the three methodological issues described above and include more detailed coverage of other health and non-health outcomes would be significant contributions for both the biomedical and social science literatures. We agree with Taylor-Robinson and colleagues that more trials would be valuable but we also believe that, based on the current evidence, policymakers who have to make decisions today should treat those infected with soil-transmitted helminths.
